# Paracoccidioidomycosis in the 21st century: Challenges and milestones

**DOI:** 10.1371/journal.pntd.0013819

**Published:** 2026-01-06

**Authors:** James Venturini, Norma Beatriz Fernandez, Priscila Marques de Macedo, Ricardo de Souza Cavalcante, Diego H. Caceres, Clayton Luiz Borges, Anderson Messias Rodrigues, Antonio Luiz Dal Bello Gasparoto, Gustavo Giusiano, Wellington Santos Fava, Marcus de Melo Teixeira, Erika Seki Kioshima, Igor Massahiro de Souza Suguiura, Angel Gonzalez, Beatriz L. Gómez, Oliver K. Clay, Renata Rebello Mendes Gomes, Adriana Pardini Vicentini, Rosely Maria Zancopé-Oliveira, Jose Guillermo Pereira Brunelli, Rosana Puccia, Eva Burger, Luciane Alarcão Dias-Melicio, Maria Jose Soares Mendes Giannini, Roxana Claudia Iquize Condori, Anamelia Lorenzetti Bocca, Maria Sueli Soares Felipe, Maria Aparecida Shikanai-Yasuda, Gil Benard, Ziadir Francisco Coutinho, Simone Schneider Weber, Rinaldo Poncio Mendes, Anamaria Mello Miranda Paniago

**Affiliations:** 1 School of Medicine, Universidade Federal de Mato Grosso do Sul, Campo Grande, Mato Grosso do Sul, Brazil; 2 Laboratorio de Micología Clínica, División Infectología, Hospital de Clínicas José de San Martín, Buenos Aires, Argentina; 3 Cátedra de Microbiología Clínica, Área Micología Clínica, Facultad de Farmacia y Bioquímica, Universidad de Buenos Aires, Buenos Aires, Argentina; 4 Fundação Oswaldo Cruz, Instituto Nacional de Infectologia Evandro Chagas, Rio de Janeiro, Rio de Janeiro, Brazil; 5 São Paulo State University, UNESP, Botucatu School of Medicine, Botucatu, São Paulo, Brazil; 6 Studies in Translational Microbiology and Emerging Diseases (MICROS) Research Group, School of Medicine and Health Sciences, Universidad del Rosario, Bogotá, Colombia; 7 Center of Expertise in Mycology, Radboudumc/Canisius Wilhelmina Hospital, Nijmegen, The Netherlands; 8 IMMY, Norman, Oklahoma, United States of America; 9 Department of Biochemistry and Molecular Biology, Universidade Federal de Goiás, Goiânia, Goiás, Brazil; 10 Departamento de Microbiologia, Imunologia e Parasitologia, Universidade Federal de São Paulo, São Paulo, São Paulo, Brazil; 11 National Institute of Science and Technology in Human Pathogenic Fungi, São Paulo, São Paulo, Brazil; 12 Department of Mycology, Instituto de Medicina Regional, Universidad Nacional del Nordeste, CONICET, Resistencia, Argentina; 13 School of Medicine, Universidade de Brasilia, Brasilia, Federal District, Brazil; 14 Department of Clinical Analyses and Biomedicine, Universidade Estadual de Maringá, Maringá, Paraná, Brazil; 15 Department of Immunology, Parasitology and General Pathology, Universidade Estadual de Londrina, Londrina, Paraná, Brazil; 16 School of Microbiology, Universidad de Antioquia, Medellín, Colombia; 17 Department of Nutrition, Universidade Federal de Sergipe, Aracaju, Sergipe, Brazil; 18 Center of Immunology, Instituto Adolfo Lutz, São Paulo, São Paulo, Brazil; 19 Centro de Especialidades Dermatológicas, Ministerio de Salud Pública y Bienestar Social de Paraguay, San Lorenzo, Paraguay; 20 Department of Microbiology and Immunology, Universidade Federal de Alfenas, Alfenas, Minas Gerais, Brazil; 21 Department of Clinical Analysis, School of Pharmaceutical Sciences, Universidade Estadual Paulista - UNESP, Araraquara, São Paulo, Brazil; 22 Caja Nacional de Salud de Bolivia/Clinica Las Panosas, Tarija, Bolivia; 23 Department of Cell Biology, University of Brasilia, Brasilia, Federal District, Brazil; 24 Postgraduate Program in Genomic Sciences and Biotechnology, Universidade Católica de Brasília, and Universidade de Brasília, Brasília, Federal District, Brazil; 25 Departamento de Infectologia e Medicina Tropical, Faculdade de Medicina, Universidade de São Paulo, São Paulo, São Paulo, Brazil; 26 Laboratorio de Investigaçao Medica em Imunologia (LIM-48), Hospital das Clinicas da Faculdade de Medicina, Universidade de São Paulo, São Paulo, São Paulo, Brazil; 27 Laboratorio de Micologia Médica (LIM-53), Instituto de Medicina Tropical, Faculdade de Medicina, Universidade de São Paulo, Sao Paulo, São Paulo, Brazil; 28 Departamento de Dermatologia, Faculdade de Medicina, Universidade de São Paulo, São Paulo, São Paulo, Brazil; 29 Fundação Oswaldo Cruz, Escola Nacional de Saúde Pública, Centro de Saúde Escola Germano Sinval Faria, Rio de Janeiro, Rio de Janeiro, Brazil; 30 School of Pharmaceutical Sciences, Food and Nutrition, Universidade Federal de Mato Grosso do Sul, Campo Grande, Mato Grosso do Sul, Brazil; University of Florida, UNITED STATES OF AMERICA

## Abstract

**Background:**

Paracoccidioidomycosis (PCM) is a neglected tropical fungal disease endemic to Latin America that predominantly affects rural and socioeconomically vulnerable communities. Despite significant morbidity, mortality, and substantial public health implications, PCM remains frequently underdiagnosed and underreported, mainly due to inadequate disease awareness and insufficient surveillance systems. This narrative review highlights recent milestones in the etiology, ecology, epidemiology, clinical manifestations, diagnosis, treatment, antifungal drugs, host–pathogen interactions, genetics, omics approaches, sequelae, and social aspects of PCM. Additionally, it identifies ongoing challenges and critical knowledge gaps for future research.

**Methods:**

A systematic retrieval of articles published between 2001 and 2025 was conducted from PubMed and the Virtual Health Library (BVS), using descriptors (“Paracoccidioidomycosis” OR “Paracoccidioides”). Duplicate records were removed through the Rayyan QCRI, and two reviewers independently evaluated the articles according to predefined thematic areas.

**Findings:**

Recent advancements have enhanced our understanding of PCM epidemiology, driven by ecological shifts and socioeconomic transformations that alter disease distribution and clinical presentation. Although substantial progress has been made in identifying and characterizing the causative agent, *Paracoccidioides* spp., challenges persist in the diagnostic process owing to limited laboratory methodologies and the absence of standardized tests. Current therapeutic options face limitations such as prolonged treatment durations, frequent drug interactions, and complicating disease management. Moreover, PCM significantly affects patients’ quality of life through persistent physical sequelae, psychological impacts, and socioeconomic consequences, including stigmatization and reduced work capacity.

**Conclusion:**

Addressing these multifaceted challenges requires integrated approaches that combine improved surveillance, enhanced diagnostic tools, novel therapeutic strategies, and targeted social support programs. Sustained collaborative research and international cooperation are essential to fill existing knowledge gaps and achieve better health outcomes for affected populations.

## Introduction

Paracoccidioidomycosis (PCM) is an endemic, geographically restricted systemic mycosis and occupational disease. The true impact of PCM remains poorly understood, mainly because PCM is not a reportable disease in most endemic regions. This policy gap contributes to its continued neglect, underdiagnosis, and underreporting, limiting accurate epidemiological tracking and understanding of its spread. In 2022, the World Health Organization (WHO) classified *Paracoccidioides* spp., the fungi responsible for PCM, as priority fungal pathogens because of their significant impact on susceptible and vulnerable populations, assigning them to the medium-priority category [1]. This review aims to highlight the milestones achieved in the understanding and management of PCM in the 21st century, including significant advancements in epidemiology, ecology, pathogen biology, clinical manifestations, fungal–host interplay, diagnostics, therapeutics, and social aspects. Additionally, it seeks to outline ongoing challenges and knowledge gaps that persist, underscoring critical areas requiring continued investigation and international collaboration to better manage this important neglected mycosis.

## Methods

### Ethics statement

No ethical approval was needed, as the study involved the analysis of previously published articles.

This article is a narrative review prepared according to the editorial guidelines of *PLOS Neglected Tropical Diseases*, which require a concise description of literature search and appraisal. We searched PubMed (https://pubmed.ncbi.nlm.nih.gov/ and the Virtual Health Library (BVS; https://bvsalud.org/, which indexes multiple regional databases (e.g., LILACS, PAHO-IRIS, BINACIS), for articles published between 2001 and 2025 using the descriptors “Paracoccidioidomycosis” OR “Paracoccidioides.” Results were managed in Rayyan QCRI (https://rayyan.ai/, duplicates were removed, and two authors independently screened the records.

The inclusion criterion was any study addressing PCM published in the 21st century (2001–2025). Selected references were critically appraised for relevance and contribution to the field, consistent with the narrative nature of this review. For organizational purposes, the literature was grouped into 12 thematic areas (etiology, ecology, epidemiology, clinical manifestations, diagnosis, treatment, antifungal drugs, host–pathogen interactions, genetics, omics approaches, sequelae, and social aspects). Articles could be classified in more than one category when appropriate, depending on the scope of their contribution. The authors then critically appraised the selected literature for quality and relevance to ensure an accurate representation of current knowledge and trends in PCM research.

## Results

The preliminary literature search identified 4,812 articles from the PubMed and BVS databases. After the removal of duplicates, 2,491 unique articles remained. Subsequent screening was conducted on the basis of predefined thematic criteria, resulting in the selection of 2,256 articles that met the inclusion criteria and were selected for qualitative synthesis in this review. These articles were categorized into twelve thematic areas as follows: genetic/genomic (*n* = 683), diagnosis (*n* = 619), clinical manifestations (*n* = 570), treatment strategies (*n* = 541), host–pathogen interactions (*n* = 316), taxonomy/phylogeny (*n* = 255), immune response (*n* = 212), pathogenesis (*n* = 154), epidemiology (*n* = 139), vaccine development (*n* = 55), ecology (*n* = 17), and social impact (*n* = 10).

### Epidemiology

In the 21st century, significant epidemiological shifts in PCM have been driven by environmental changes, the expansion of agricultural frontiers, modernized agricultural practices, and socioeconomic factors. This temporal-geographical dynamic has influenced not only the distribution of PCM cases but also the clinical manifestations of the disease, which vary significantly among different populations. The chronic form of PCM remains predominant, accounting for approximately 80% of cases, and is primarily observed among adult rural male workers [2]. While its incidence has declined in areas with more mechanized and modernized agriculture, the number of cases has risen in regions experiencing new agricultural frontiers. A similar trend is observed in the incidence of the PCM acute/subacute form, which has decreased in areas where child labor has been reduced but has increased in other regions [3].

This rise is particularly pronounced in endemic areas characterized by poverty and malnutrition, which adversely affect children and increase their susceptibility to the disease. Furthermore, environmental disruptions, including road construction, hydroelectric dam projects, and agricultural expansion, have been identified as contributing factors to the increase in acute/subacute form [4–10].

**Fig 1:**
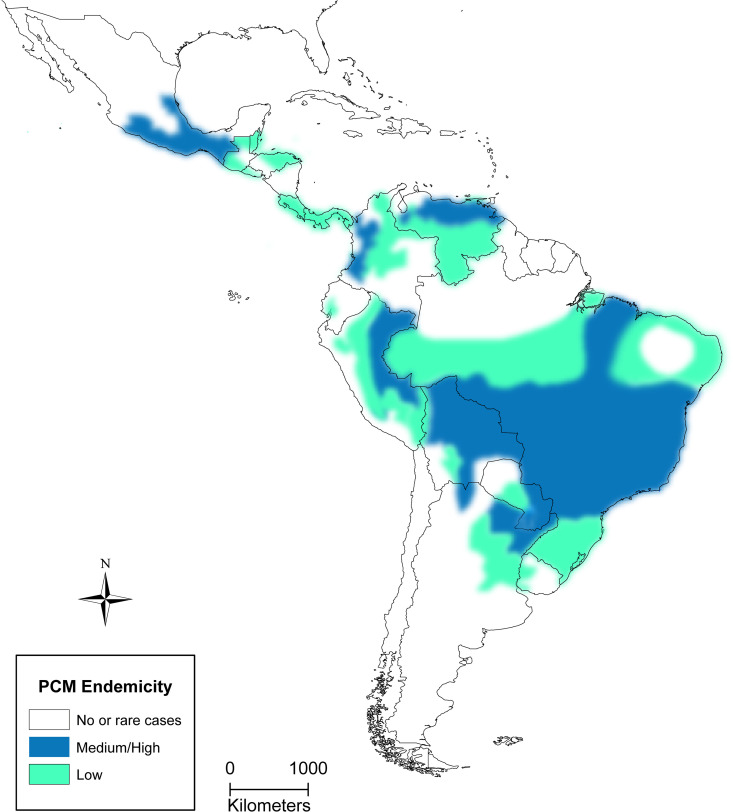
Geographical distribution and endemicity levels of paracoccidioidomycosis (PCM) in the Americas. Areas are categorized as no or sporadic cases (white), low endemicity (light green/teal), and medium/high endemicity (blue). The classification was based on expert consensus (modified Delphi process) conducted during the International Conference on Paracoccidioidomycosis (PCM XXI, Campo Grande, Brazil, 2024), integrating published data and local expert knowledge. This map was created using ArcGIS software by Esri. ArcGIS and Arc-Map are the intellectual property of Esri and are used herein under license. Copyright Esri. All rights reserved. For more information about Esri software, please visit www.esri.com. Basemap: GADM layers (https://gadm.org/download_country.html) under CC BY 4.0 license.

PCM is geographically confined to the region from Mexico to Argentina, with Brazil having the highest prevalence, accounting for 80% of the cases reported in Latin America. However, the true endemicity of PCM remains unclear. The map of PCM endemicity (Fig 1) was developed through a modified Delphi consensus process [11] conducted during the International Conference on Paracoccidioidomycosis (PCM XXI, Campo Grande, Brazil, December 2024). Experts from endemic countries reviewed published data, surveillance reports, and local epidemiological knowledge to reach consensus on endemicity levels. A detailed description of the methodology is provided in the S1 File.

In Brazil, the expansion of the agro-industry and agro-energy sectors has played a crucial role in shaping the epidemiological dynamics of PCM. In the state of Rondônia, situated within the southern Amazon biome and a hotspot for PCM, a substantial increase in cases has been observed among indigenous ethnic groups since the 1990s [12]. The southern region, which is known for extensive family farming, has been identified as the region with the highest incidence of PCM, with a mortality rate of six deaths per million people. Moreover, new high-endemicity regions, such as MATOPIBA (Maranhão, Tocantins, Piauí, and Bahia states), have been observed, characterized by an increased occurrence of autochthonous PCM cases in urban areas and increasing coinfections with *Paracoccidioides* species [12–14]. In south-central Brazil, recent data also indicate a reduction in both acute/subacute and chronic forms, particularly associated with the modernization of agricultural practices. This contrasts with regions where incidence remains higher due to persistent socioeconomic vulnerabilities [3]. Conversely, PCM remains one of the most important mycoses in the region, showing a higher mortality rate than other fungal infections, as documented in Paraná State between 2007 and 2020 [15]. To contextualize the broader impact, the overall mortality rate in Brazil stands at 1.5 per million people [16], whereas the hospitalization rate is 4.3 per million, covering 35% of the country [6].

The prevalence and incidence of PCM remain largely unknown in other countries of the endemic regions. Nevertheless, chronic form cases are mostly reported among adult rural workers across the region. For example, 479 cumulative cases have been documented in Paraguay since 1935 [17]. Although Bolivia is considered a country of medium to high endemicity in some regions, the number of documented cases in the literature remains low [18,19]. In Argentina, the epidemiological landscape is divided into two distinct geographical areas: northeastern and northwestern regions. In northwestern Argentina, where caloric-protein undernutrition affects 10%–37% of the population, patients are typically younger and exhibit more severe disease manifestations, including gastrointestinal alterations, splenomegaly, and ascites. Conversely, in northeastern Argentina, patients are predominantly older and more frequently exhibit cutaneous lesions of the disease [20]. In Venezuela, a retrospective study analyzing 31,081 clinical records over 65 years (1954–2019) confirmed 745 PCM cases, predominantly chronic in form (90%), affecting mainly male rural and construction workers aged 41–60 years. Miranda State showed the highest prevalence, with lungs, skin, and oral mucosa as the most frequently involved sites. Molecular analysis identified *P. venezuelensis* as the dominant species in the country [21].

The scarcity of epidemiological information is partly explained by the fact that PCM remains a non-notifiable disease across all Latin American countries. Expanding awareness strategies, encompassing digital patient networks, telehealth support, and patient advocacy, could further enhance the integration of notification frameworks into public health policies and reinforce their urgency as a reportable disease. Harmonizing disease classification and notification systems across endemic regions is essential for improving data reliability and guiding more effective public health interventions.

In this context, another critical barrier is the current International Classification of Diseases, 10th Revision (ICD-10), which does not align with the standard clinical classification of PCM that distinguishes between acute/subacute, chronic, mixed, and residual forms. Such misalignment hampers the accurate interpretation of public health data. A practical step to reduce this discrepancy would be to adopt this consensus clinical classification, initially proposed in Medellín (1986) and endorsed in the Brazilian Guidelines [4], as the basis for coding. In addition, expanding the ICD-10 to include items beyond the pulmonary and disseminated forms currently listed should be considered to cover and highlight other important sites of organ involvement.

### Etiological agents and ecology

Among the Onygenales, the family Ajellomycetaceae includes species of the genus *Paracoccidioides*, which are the fungal pathogens responsible for PCM. This genus includes the *P. brasiliensis* species complex—composed of *P. brasiliensis sensu scrictu* (clade Sb1a-Sb1b), *P. americana* (PS2), *P. restrepiensis* (PS3), and *P. venezuelensis* (PS4)—as well as the distinct species *P. lutzii* [22,23]. In addition to these cultivable species, the genus also includes non-cultivable species such as *P. ceti* and *P. lobogeorgii* (formerly *Lacazia loboi*), which causes chronic subcutaneous infections in dolphins and humans, respectively [24].

Recent developments in the taxonomy and nomenclature of medically important fungi have led to scientifically robust and necessary revisions, grounded in molecular phylogenetics. At the same time, such changes may carry practical consequences for clinical and public health practice, including the adjustment of diagnostic tests, reinterpretation of surveillance data, and revision of textbooks and clinical manuals (e.g., candidiasis) [25–29]. These implications do not represent opposition to taxonomic advances but rather highlight the need for coordinated strategies to ensure their effective integration into diagnostic, surveillance, and educational frameworks.

The soil is believed to be the natural habitat of cultivable *Paracoccidioides*. These *Paracoccidioides* species are dimorphic, and inhabit two distinct ecological niches by adopting different morphological forms. In the environment, they exist as a mycelial phase, which thrives at temperatures of 22–25°C. Hosts typically become infected by inhaling hyphal fragments or conidia from the soil produced by this mycelial form. Once inside the host, the fungus transitions to a yeast phase, adapted to body temperatures of 35–37°C (S2 File). Specific selection factors govern the development and persistence of each phase. *Paracoccidioides* spp. exemplify this biological strategy, characterized by their ability to dynamically adapt and alternate between the mycelial and yeast forms throughout their life cycle [4,7,30]. The environmental phase of *Paracoccidioides* presents variable infective structures among species that are dependent on environmental conditions such as soil composition and air humidity. In contrast, the infective phase within the host is characterized by a prolonged period of infection and disease latency.

The geographic distribution of *Paracoccidioides* species in the Americas reveals a complex and evolving pattern shaped by both ecological factors and advancements in molecular diagnostics [31]. *P. brasiliensis sensu stricto* is the most widespread species and is found across much of South America. In contrast, species such as *P. americana*, *P. restrepiensis*, and *P. venezuelensis* exhibit more localized or initially restricted ranges, Brazil and Venezuela for *P. americana*, Colombia for *P. restrepiensis*, and Venezuela for *P. venezuelensis,* although molecular studies have revealed broader, often overlapping distributions. *P. lutzii*, which was originally confined to central and northern Brazil, is now emerging in new regions such as southeastern Brazil and Ecuador [5,32,33]. The increasing detection of multiple species in single regions and the emergence of species in previously unrecognized areas underscore the dynamic biogeography influenced by environmental disruption, human migration, and improved surveillance [7].

Several surveillance strategies have been employed to detect the presence of the *Paracoccidioides* genus in the environment. These strategies have successfully identified the fungus not only in soil and animals but also in unusual habitats, such as water, as evidenced by *Paracoccidioides* infection in fish [34]. A breakthrough in metagenomics, applied to the microbiome, has facilitated an understanding of fungal presence in the environment and its potential impacts on the population. These investigative tools have been instrumental in detecting *Paracoccidioides* in diverse geographical regions, including the Cerrado ecoregion—a vast tropical savannah in Brazil known for its enormous biodiversity. Additional findings include soil from abandoned houses, armadillo habitats, areas with owl feces, and sugarcane and coffee plantations [35,36].

The natural occurrence of PCM in animals has been reported much less frequently than in humans [37]. Evidence of infection by cultivable *Paracoccidioides* species has been described in several domestic and wild animals, including dogs, cats, pigs, goats, sheep, horses, cattle, chickens, rabbits, monkeys, small rodents, among others. However, the detection of PCM agents through isolation and genomic methods is well documented in armadillos, which are therefore considered the main natural reservoirs of the fungi [38]. On the other hand, clinical manifestations of the disease have been reported in only a few species, especially in dogs and monkeys [38–40]. Research employing diverse animal models, including hamsters, guinea pigs, dogs, mice, rats, and bats, has been instrumental in enhancing our understanding of *Paracoccidioides* biology and pathogenesis.

Taken together, these ecological and experimental findings highlight the complex interaction between *Paracoccidioides* species, their hosts, and the environment. Understanding the ecological niches and environmental reservoirs of *Paracoccidioides* is essential not only for identifying sources of infection but also for anticipating how environmental changes may influence its distribution and transmission dynamics. Multiple climate models and observational studies indicate that global warming alters the tropical Pacific background state, affecting the El Niño–Southern Oscillation system [41]. More frequent and intense El Niño events increase soil water storage and air humidity in endemic regions, conditions that may favor not only the survival but also the proliferation of *Paracoccidioides* in the soil and the dispersion of its conidia. Clusters of acute/subacute PCM cases have been temporally and spatially associated with moderate and strong El Niño episodes, suggesting that atypical environmental conditions precede increases in disease incidence [10,42,43]. This fits within a broader framework where climate change is expected to reshape fungal ecology and distribution [44].

### Clinical challenges

#### Clinical forms.

To date, the incubation period of PCM cannot be accurately determined because it is not possible to identify when the fungus is inhaled. Children, adolescents, and young adults present short duration of symptomatology (approximately two months), involving organs in the reticuloendothelial system, such as the lymph nodes, liver, spleen, and bone marrow; the lungs are rarely affected (approximately 5%), despite being the fungal portal of entry. This clinical presentation was named the **acute/subacute form**, and the age of the patients suggested a short incubation period (S3 File). It was classified into moderate and severe. Some patients over 30 years of age who presented with the acute/subacute form also had an immunosuppressive comorbidity, suggesting that this clinical presentation may be associated with an impaired ability to mount an effective cellular immune response against the fungus. The prevalence of acute/subacute form varies across regions, ranging from 5% to 25% of PCM cases [4,45]. An increased prevalence was observed in some Brazilian areas due to climate alterations (El Niño) and anthropogenic actions in the environment, as previously mentioned [10,43,46]. In contrast, the prevalence has decreased in areas where child labor has been prohibited [3]. The male:female ratio of the acute/subacute form is 1.7:1.0 [47].

Most PCM patients are at least 30 years old and present with insidious disease with long duration (symptomatology usually more than four months, many of them over six months), high prevalence of pulmonary involvement, practically a rule, and frequent skin and mucous membranes of the upper aerodigestive tract lesions (S3 File). Lymph node enlargement can be present, but it predominates in the cervical and submandibular chains and is of the inflammatory nonsuppurative type, i.e., with a diameter <2 cm, without signs of suppuration. This clinical presentation was named the **chronic form** and was classified according to severity into mild, moderate, and severe. The chronic form accounts for 75%–95% of the cases. The demonstration of the paracoccidioidal primary complex in autopsies of patients with other diseases, the report of Brazilian patients who moved from rural areas many years before diagnosis, and PCM cases in patients from Europe and Asia, who had lived in South American countries, from where they left many decades before, suggested that the disease was caused by reactivation of latent foci [4,48,49]. The involvement of other organs has been reported, and the male:female ratio of the chronic form is 22:01 [47].

A study of PCM-AIDS comorbidity revealed that the same patient can present clinical manifestations compatible with acute/subacute and chronic forms, such as those in the lungs, upper aerodigestive tract, liver, spleen, and lymph nodes. It was suggested that the name **mixed form** for this type of clinical presentation [49]. However, atypical clinical presentations have long been reported in patients with concomitant cancer and PCM [50] initially recognized by pioneer clinicians dedicated to the study of this mycosis. Therefore, PCM patients presenting with mixed or atypical forms should be carefully investigated to exclude any underlying immunosuppressive condition.

Although rare, some patients present with involvement of a single organ, uncommon among those affected by PCM, and its regional lymphatic system, which cannot be classified under the previously defined clinical forms. This presentation has therefore been termed the **isolated organic form** [48,49]. The involvement of the lymphatic system in this clinical form is localized and often detected only through histopathological examination. To define this clinical form, it is first necessary to exclude the involvement of organs that are classically affected by PCM, such as the lungs, skin, mucous membranes, lymph nodes, liver, spleen, bone marrow, adrenals, central nervous system, bones, and genitals.

Many patients present with sequelae after successful treatment, such as microstomia, hoarseness, intestinal occlusion or subocclusion, Addison’s syndrome, pulmonary fibrosis, and emphysema. These clinical presentations are referred to as **residual forms** or **sequelae**. This clinical form should be highlighted because surgical or clinical treatment must be carried out and, in many cases, leads to a decrease in job capacity or retirement, with important social consequences [49,51]. As pulmonary involvement is very common in the chronic form, and the extent of lung lesions is variable, their severity was classified radiographically and tomographically, for the first time, both at diagnosis (active disease) and after successful treatment (sequelae) [52]. They can lead to chronic respiratory failure followed by pulmonary hypertension and heart failure, and increase the risk of secondary pneumonia.

Thus, the classification of the clinical forms, including their severity, was improved in the 21st century.

#### Clinical presentations in challenging scenarios.

Some scenarios continue to be a challenge owing to diagnostic difficulties and clinical management, as presented below.

***Central nervous system (CNS).*** The prevalence of CNS involvement in PCM is generally considered low, ranging between 1.0% and 3.5% in older studies, with more recent Brazilian data reporting rates ranging from 3.8% to 13.9% in clinical series [45,53–55]. CNS involvement affects both acute/subacute and chronic forms similarly. Encephalic lesions are predominant (up to ~80% of cases in historical cohorts), especially in the cerebral hemispheres, cerebellum, and brainstem, followed by meningeal and spinal cord manifestations. The most common clinical manifestations include headache, seizures, and focal neurological signs [45,53–55]. The identification of *Paracoccidioides* spp. or specific antibodies by precipitation tests is usually negative in Cen Fluid. The diagnosis is confirmed by cerebrospinal fluid determination of antibodies or antigens or by the detection of the specific antigen gp43 or anti-gp43 via a latex test [56]. Imaging-based methods present high sensitivity, despite low specificity, but are crucial for confirming CNS involvement [57]. Intramedullary spinal cord involvement has rarely been observed, usually as a reported case; the sequelae are frequent and severe [58]. The management of CNS involvement in PCM is challenging and requires antifungal agents with good CNS penetration. Trimethoprim-sulfamethoxazole (TMP-SMX) is considered the first-line treatment, especially for moderate or severe cases, as monotherapy. In patients with severe disease or refractory cases, amphotericin B is a treatment option, with preference given to its liposomal formulation due to better tolerability and reduced nephrotoxicity. TMP-SMX in combination with fluconazole (FLC) is also another treatment option. This combination has shown favorable outcomes in reported cases [58–60]. High-dose FLC has also been employed as monotherapy, especially in patients with renal impairment or contraindications to TMP-SMX or amphotericin B [61,62]. Intravenous voriconazole has been reported as a treatment option in an isolated case report [60]. It is suggested that in the active neuroparacoccidioidomycosis, the role of this azole or other combinations previously mentioned be established in comparative multicenter studies. However, its use remains extremely limited in neuroparacoccidioidomycosis due to high cost, significant drug interactions, and unpredictable oral bioavailability, which limit its utility as a long-term maintenance therapy. In selected cases, surgical interventions such as ventricular drainage, lesion excision, or decompression may be required.

***The skin and mucous membranes.*** Cutaneous manifestations of PCM are indicative of systemic dissemination and may precede respiratory symptoms, particularly in the acute/subacute form. Their frequency is high across all age groups, with reported values ranging from approximately 47% to over 70%. Lesions are most frequently located on the face, followed by the trunk and limbs. The most common morphological presentation includes ulcerative and ulcerovegetative lesions, while infiltrative, sarcoid-like, verrucous, and mixed lesions are also observed. Owing to their clinical pleomorphism, cutaneous lesions may mimic other granulomatous or neoplastic diseases, such as sarcoidosis, tuberculosis, leishmaniasis, and other systemic mycoses [49]. Importantly, skin lesions are often valuable for mycological diagnosis, offering accessible material for direct visualization and culture of the etiological agent [61]. In addition, hyperpigmentation of the skin suggests the involvement of the adrenal glands. In some cases, skin lesions progress to sequelae after treatment [63]. Immunocompromised patients present diffuse and atypical skin lesions, hampering diagnostic suspicion. The involvement of the oral mucosa results in severe stomatitis, with ulcers and vegetating lesions presenting purulent secretions rich in fungal cells. Patients with skin and oral and/or nasal mucosal involvement present with stigmatizing aesthetic alterations. The skin, as a route of paracoccidioidal infection, has rarely been reported as a result of direct inoculation via laboratory accidents or trauma from environmental sources [64].

***Pregnancy.*** The appearance of clinical manifestations during pregnancy may arise from direct progression from the primary complex or from endogenous reinfection due to the decreased immune response presented by these patients. They usually manifest as severe acute/subacute form with diffuse skin lesions. Although maternal–fetal transmission has not been reported, placental involvement may affect fetal circulation, leading to small and low-birth-weight newborns. The safe antifungal compounds for treatment are AmB and L-AmB, which diffuse to the fetus but are not teratogenic; however, their intravenous administration requires hospitalization for a long period of time. The azole derivatives are teratogenic, well documented for FLC, and high risk for ketoconazole and itraconazole (ITC). The TMP-SMZ combination presents a low risk of teratogenicity during the first trimester of pregnancy, and the risk of kernicterus—it must be discontinued at the end of pregnancy. The severity of the disease and the potential sequelae are also noteworthy [4,65].

***Solid tumors association.*** Although the first reports of an association between PCM and solid tumors date back to the early 20th century, this comorbidity remains underexplored in the literature. The available clinical data suggest that co-occurrence is more common with solid malignancies than with hematological malignancies, predominantly affecting patients over 50 years of age with the chronic form. In most cases, lesions of both diseases are identified at the same anatomical site, with PCM typically preceding the diagnosis of cancer or being diagnosed concomitantly. The most commonly affected systems include the respiratory and digestive tracts, followed by the skin, genitourinary tract, larynx, and endocrine glands. Several hypotheses have been proposed to explain this association, including chronic inflammation, fibrotic remodeling, exposure to tobacco and alcohol, and potential genetic alterations. Given the possibility of PCM reactivation years after discontinuing antifungal therapy, especially in the presence of neoplasms, and the reduced sensitivity of immunodiffusion assays in these scenarios, this differential diagnosis must be considered in endemic regions. Diagnostic confirmation relies on histopathological analysis, fungal culture, and serological testing, such as the dot blot assay. In cases requiring antifungal and antineoplastic therapies, potential drug interactions must be carefully evaluated during treatment planning [50,66,67].

***Malabsorption syndrome.*** This complication was highlighted by the observation of a confirmed case of the severe acute/subacute form of PCM with malabsorption syndrome. Sequential abdominal scintigraphy using 99mTc-albumin demonstrated progressive protein-losing enteropathy, supporting the diagnosis [68]. The management of malabsorption syndrome in PCM should involve the monitoring of nutritional biomarkers [57], including i) fat-soluble nutrients, such as vitamins A, D, E, and K, as well as serum cholesterol, and ii) water-soluble nutrients, such as iron (hemoglobin, ferritin, transferrin saturation), vitamin B12, plasma zinc, and serum albumin. Nutritional support may include i) a high-protein diet or whey protein supplementation; ii) restriction of long-chain fatty acids; iii) oral rehydration solutions or intravenous fluid replacement; iv) temporary exclusion of fermentable oligosaccharides, disaccharides, monosaccharides, and polyols during diarrhea episodes; and v) targeted nutrient repletion with monitoring to avoid toxicity. These measures, although not systematically retrieved in our review, are grounded in clinical reasoning and adapted from general management strategies for protein-losing enteropathies [69,70].

***AIDS association.*** Since the beginning of the 21st century, several PCM reference centers have reported their own experience with HIV–PCM patients [71–75]. The relatively small number of patients with this association was initially attributed to the different and, at least in the early years, non-overlapping epidemiological profiles of the infections (“rural” versus “urban”). However, this distinction no longer holds true. Another hypothesis proposed to explain the rarity of PCM–AIDS co-infection, also suggested at the beginning of the AIDS epidemic, was that the frequent use of prophylactic therapy with either ITC (e.g., for oral candidiasis) or cotrimoxazole (CMX) (e.g., for toxoplasmosis), both drugs with anti-*Paracoccidioides* activity, could have exerted a protective effect. Nevertheless, the occurrence of PCM in patients under such prophylaxis weakened this assumption. Therefore, a consistent explanation for the persistently low number of HIV–PCM cases (compared, for example, with the much higher number of HIV–histoplasmosis and HIV–cryptococcosis cases in Brazil) is still lacking and should be further investigated. Major findings from studies published over the past two decades indicate that, compared with the early years of the AIDS epidemic, the prognosis of HIV–PCM patients has improved considerably. Most exhibit favorable initial responses to conventional PCM treatment when the mycosis is diagnosed at early stages, even though the majority present with low CD4 cell counts (<200 cells/µL). Only rarely do patients have normal or near-normal CD4 counts, indicating that PCM most often behaves as an opportunistic infection. In fact, in approximately half of the reported cases, PCM was the opportunistic infection that led to the diagnosis of AIDS, while the other half occurred in patients with known HIV infection. Even in highly endemic areas for PCM, the proportion of AIDS patients with PCM remains very low (1.4%); however, among patients with PCM, 5.2% were HIV-coinfected [75]. Deaths were mostly related to opportunistic infections other than PCM.

Importantly, mycological examination plays a major role in the diagnosis of HIV–PCM cases due to the high fungal burden of lesions, leading to more frequent laboratory confirmation than in non-coinfected patients, in whom serological diagnosis usually predominates. Data reported up to now indicate that approximately 70% of co-infected patients display detectable antibody responses in conventional serological tests [76]. Therefore, AIDS patients with suspected PCM infection may require additional diagnostic tools beyond serology in their clinical investigation.

***Tuberculosis association.*** When PCM and tuberculosis coexist, the main challenge is to establish a timely and accurate diagnosis to ensure appropriate treatment. This requires a detailed clinical history, including place of residence, travel, and occupational exposures. The two diseases differ in their respiratory manifestations: PCM often shows clinic-radiological dissociation with mixed middle- and lower-lobe infiltrates, whereas tuberculosis typically involves the upper and posterior lung segments, frequently with cavitation. Mucocutaneous lesions are more common in PCM, whereas lymphadenopathy with node hypertrophy may occur in both. Notably, concomitant infection is difficult to manage because ITC, the first-line antifungal for PCM, interacts with rifampicin, a key drug in the standard antituberculosis regimen, markedly reducing ITC serum levels and reducing the efficacy of antifungal treatment [4].

***Sequelae.*** Another important challenge is the sequelae observed after efficacious treatment, which constitute a clinical presentation and often affect productivity or lead to early retirement [48,49]. The sequelae are due to fibrosis observed in several organs, which is associated with emphysema in the lungs. The predominance of the chronic form of PCM, along with its frequent pulmonary involvement, often results in posttreatment sequelae such as fibrosis and emphysema, which can limit patients’ functional capacity during their most productive years and negatively impact quality of life. On the basis of previous experimental studies [77,78], a proposal for a randomized multicenter clinical trial involving the use of pentoxifylline in combination with ITC was proposed, and its general lines were approved. The final design of the study should be sent to different services and conducted in the coming years.

***COVID-19 infection.*** Infection by SARS-CoV-2 has markedly increased the risk of secondary fungal infections, including aspergillosis, mucormycosis, invasive candidiasis, and outbreaks of *Candidozyma auris* (formerly *Candida auris*), particularly among critically ill patients. Severe COVID-19 disrupts immunological homeostasis, impairing antifungal defenses and potentially exacerbating the severity of PCM, which may present as mixed or disseminated clinical forms. Moreover, the use of corticosteroids for COVID-19 treatment further suppresses host immunity, compounding this effect. Despite these risks, only a few cases of PCM associated with severe COVID-19 were documented during the pandemic. This low number likely reflects the healthcare system overload caused by the pandemic and the population’s reduced access to medical care due to social isolation measures. Such circumstances may have delayed PCM diagnosis, favoring disease progression in both its acute/subacute and chronic forms and contributing to unfavorable clinical outcomes, including higher mortality rates and sequelae [79,80].

### *Paracoccidioides*–host interaction: Overview on recent findings and perspectives

Significant advances in understanding the intricate dynamics of the *Paracoccidioides*–host interaction have been achieved in the 21st century, substantially improving the understanding of the disease’s pathogenesis. From the fungal perspective, pivotal discoveries include the characterization of extracellular vesicles secreted by *Paracoccidioides* spp. and their roles in immune modulation and the expression of fungal virulence traits [81–84]. Recent findings also highlight the role of biofilm formation by *Paracoccidioides* spp., demonstrating its importance in chronic infection persistence, resistance to antifungal drugs, and evasion of host immune defenses [85]. Advanced investigations into the metabolic plasticity of *Paracoccidioides* spp. have revealed critical adaptive metabolic pathways employed by the fungus to respond to environmental stressors within the host microenvironment [86]. Genomic, transcriptomic, and proteomic studies have elucidated complex virulence factors and gene expression regulatory mechanisms, providing insights into the adaptive strategies employed by the fungus to evade host defenses and ensure persistent infection [87–93].

On the host side, considerable progress has been made in consolidating knowledge about adaptive immune mechanisms involved in PCM pathogenesis, where *Paracoccidoides* antigen-specific cellular immune response impairment is observed. There has been an expanded characterization of innate immune responses, including the role of neutrophil extracellular traps, an important antimicrobial defense mechanism produced by neutrophils [94]. In addition, the genetic components underlying susceptibility to PCM have been explored, revealing associations between single-nucleotide variants [95] and increased disease risk for selected targets, as well as reports of inborn errors of immunity predisposing individuals to atypical and/or severe PCM [96,97].

**Fig 2:**
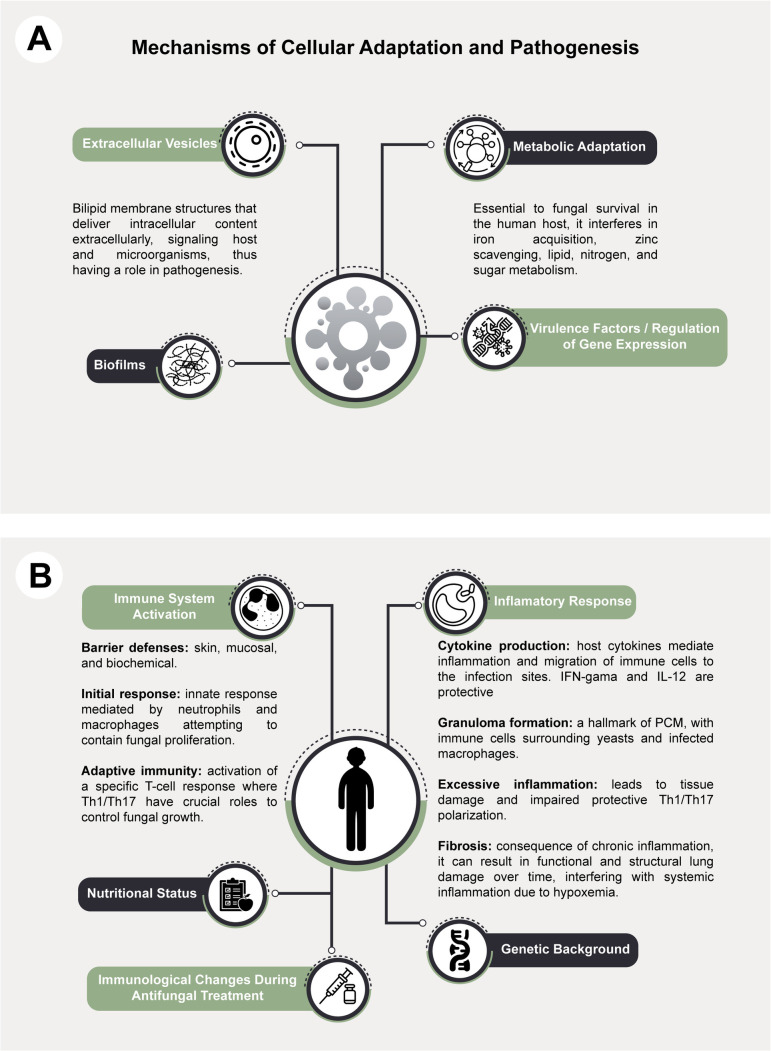
*Paracoccidioides*–host interaction. **(A)** Host responses related to the immune system activation and inflammatory response. Note that the immune system direct activation of the defense barriers, innate, and adaptative responses are influenced by the nutritional status, since malnutrition negatively impacts the immune system effectiveness. The immunological response can also change during antifungal treatment, when the specific T-cell response tends to recover, followed by persistent low-grade inflammation. Considering the inflammatory response, it can be impacted by the genetic background, specifically by single-nucleotide polymorphisms (SNPs) (IL-10, CTLA4, DC-SIGN, VDR, NLRP1) and innate immune errors, IIE (STAT4, CD40L, IL-12Rbeta1, CARD9). **(B)** Mechanisms of fungal adaptation and pathogenesis, pointing out extracellular vesicles, biofilms, metabolic adaptation, and other virulence factors. Biofilms have been detected in pulmonary epithelial cells and keratinocytes; they protect the fungi against the immune attack and antifungal drugs. Besides, microbial associations with *Candida albicans*, *Mycobacterium tuberculosis*, and *Klebsiella pneumoniae* negatively impact disease severity and treatment. Virulence factors include Hsp90, proteases, elastase, formamidase, adhesins gp43 and enolase, anti-oxidant superoxide dismutase, and cell wall alpha-1,3-glucan; many are moonlight proteins. The figure was created by us using Adobe Illustrator. All icons included in the figure, except for the *Paracoccidioides* spp. illustration (created by the authors), were obtained from The Noun Project (https://thenounproject.com/), under the Creative Commons Attribution 3.0 (CC BY 3.0) license.

Additionally, several immune alterations that occur during and after antifungal treatment have been documented and are associated with sequelae, particularly pulmonary fibrosis [98,99]. These findings emphasize the importance of longitudinal immune monitoring in patients receiving antifungal therapy, aiming to improve clinical outcomes and mitigate persistent tissue damage. Collectively, these significant 21st-century milestones provide a comprehensive understanding of the host–*Paracoccidioides* interplay, identifying pivotal avenues for future research and therapeutic innovation (Fig 2A and 2B).

In addition to the advances already discussed, future research should prioritize the implementation of cutting-edge methodologies to dissect the *Paracoccidioides*–host interface further. In recent years, the larvae of *Galleria mellonella* have been increasingly used as an alternative infection model, in line with the 3Rs principle (replacement, reduction, refinement). This insect model shares several structural and functional similarities with mammalian innate immunity, including phagocytosis and the production of antimicrobial peptides and reactive oxygen species [100]. As such, it provides a useful and ethically advantageous system to investigate host–pathogen interactions and to test antifungal compounds before validation in vertebrate models [101,102]. These models complement mammalian systems, enabling high-throughput screening of mutant strains or therapeutic candidates [48,103–105]. In addition, single-cell RNA sequencing (scRNA-seq), spatial transcriptomics, proteomics, metabolomics, and chemoproteomics, together with 3D organoid models, may provide unprecedented resolution in identifying host immune heterogeneity and localized fungal persistence. The integration of CRISPR-based functional genomics in both fungal and host cells also holds promise for identifying novel pathways involved in immune evasion and virulence.

### Laboratory diagnosis

**Fig 3:**
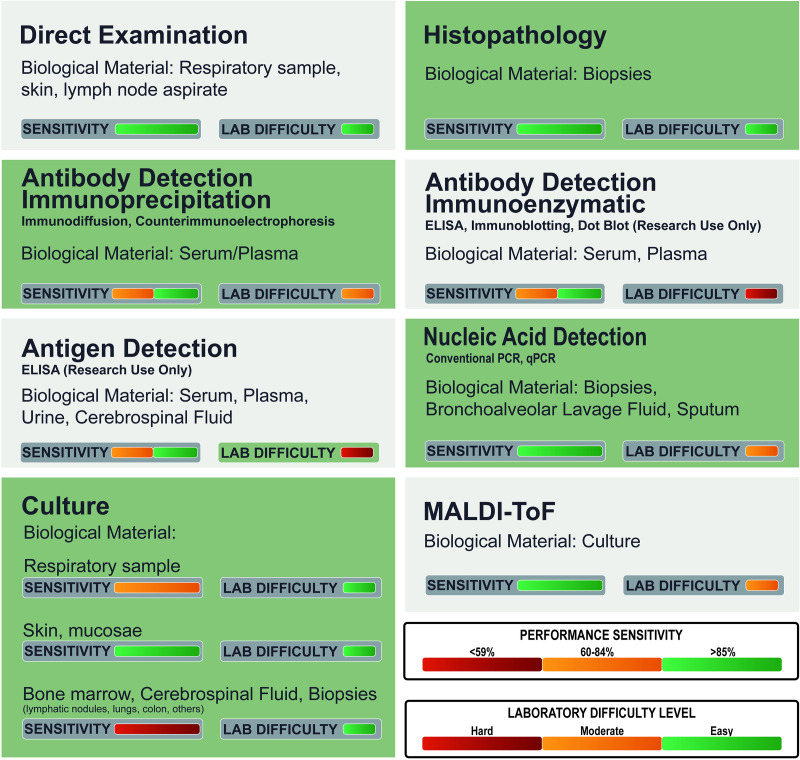
Comparative overview of the main diagnostic tools for paracoccidioidomycosis (PCM), detailing their sensitivity and laboratory difficulty. Each diagnostic method is presented with its typically required biological material(s). The performance sensitivity is visually rated via color-coded horizontal bars (green: >85%; orange: 60%–84%; red: <59%), and the laboratory difficulty is similarly displayed (green: easy; orange: moderate; red: hard). The figure was created by us using Adobe Illustrator/), under the Creative Commons Attribution 3.0 (CC BY 3.0) license.

The diagnosis of PCM relies on three fundamental pillars: clinical evaluation, epidemiological assessment, and laboratory studies, including routine direct examination, histopathology, culture-based techniques, immunodiagnostic tests (antibody and antigen detection), and molecular assays (Fig 3 and Table 1). Laboratory diagnosis is essential to ensure timely treatment, prevent severe sequelae, and reduce the risk of discontinuing treatment.

**Table 1:** Overview of the main limitations of diagnostic tools for PCM.

Method	Main limitation	References
Direct Examination^1^	Requires laboratory technician training	[48]
Histopathology^2^	Requires specialized medical training for proper specimen collection and pathologist expertise. Longer turnaround time.	[126]
Culture	Culture incubation time required (4–6 weeks) Mycelial form—Biosafety Level 3	[48]
Antibody Detection—Immunoprecipitation assays: ID, CIEF	Sensitivity depends on the endemic area or the species High rate of interlaboratorial variability Reduced sensitivity in immunosuppressed hosts Nonstandardized Ag preparation (variety of isolates and protocols)^3^^,^^4^	[111,127]
Antibody Detection—Agglutination assay: Latex	Not routinely used. Exclusively in-house assay.	[56,128]
Antibody Detection—Immuno-enzymatic assays: ELISA, Immunoblotting, and Dot Blot.	Not routinely used. Performed in a few centers. Exclusively in-house assays Nonstandardized recombinant Ag/protein^3^^,^^4^	[127]
Antigen Detection: ELISA and Latex assay	Not routinely used High cross-reactivity. Nonstandardized Ab^3^^,^^4^	[56]
Nucleic Acid Detection: Conventional PCR, Nested PCR, qPCR, Duplex PCR, Multiplex PCR	Not routinely used Exclusively in-house assays No multicentric standardization Variety of DNA extraction protocols, molecular targets, and PCR methodologies	[31,33,118,119]
MALDI-ToF MS	Requires culture. Limited database for cryptic species. Mycelial form—Biosafety Level 3	[109]

^1^KOH, Calcofluor, Gomori stains.

^2^Gomori methenamine-silver, hematoxylin and eosin, and periodic acid-Schiff stains were used.

^3^In-house tests.

^4^Production varies by center.

**Abbreviations:** Ag, antigen; Ab, antibody; ID, immunodiffusion; CIEF, counterimmunoelectrophoresis; ELISA, enzyme-linked immunosorbent assay; PCR, polymerase chain reaction; qPCR, quantitative PCR; MALDI-ToF MS, matrix-assisted laser desorption/ionization time-of-flight mass spectrometry.

In practice, the clinical specimens most commonly used for direct microscopic examination and fungal culture are sputum, biopsy material from oropharyngeal and cutaneous lesions, and lymph node aspirates, since these sites are easily accessible and frequently involved. When such sites are not affected, additional specimens may be required, including bronchoalveolar lavage fluid, cerebrospinal fluid, bone marrow aspirates, and biopsies of lymph nodes or deep tissues such as the lungs, adrenal glands, gastrointestinal tract, and central nervous system. These procedures, however, usually involve more invasive techniques or surgery. Histopathological analysis can be performed on any biopsied tissue or bronchoalveolar lavage sample, while antibody detection is carried out in serum or plasma, and antigen detection in urine, bronchoalveolar lavage, and cerebrospinal fluid [106].

Beyond these conventional approaches, a range of laboratory methodologies, including Matrix-Assisted Laser Desorption/Ionization Time-of-Flight Mass Spectrometry (MALDI-ToF MS) have been used, especially for research purposes [4,33,49,107–110]. Nevertheless, in the 21st century, no major advances have been achieved in PCM diagnostics, as no commercial methods are currently available. High costs, lack of standardization, limited reagent availability, and the need for specialized laboratories continue to hinder access within public healthcare systems. These challenges are particularly evident in antigen production, where issues of specificity, stability, repeatability, and reproducibility remain unresolved [111]. To overcome these limitations, potential strategies include strengthening regional reference centers to ensure shorter diagnostic times and more equitable access. In parallel, fostering the development and distribution of serological and molecular methods by the diagnostic industry could further expand availability and promote greater sustainability of PCM diagnosis.

Finally, omics sciences have facilitated the identification of biomolecules, genes, proteins, and metabolites, thereby advancing the understanding of candidate antigens of *Paracoccidioides* spp. Advancements in omics technology can potentially address challenges associated with PCM diagnosis, particularly those that arise during the development of immunological tests, such as cross-reactions with other fungal pathogens [111,112].

The advent of genomics in recent decades has significantly improved the efficiency of fungal DNA detection [113]. The glycoprotein gp43, which has been extensively characterized as a specific *Paracoccidioides* antigen in numerous serological tests [114], is still highly valuable for detecting PCM caused by the *P. brasiliensis* complex, but not by *P. lutzii* isolates [115]. Consequently, the development of molecular tests for diagnosing PCM requires strategies that consider species diversity in accordance with taxonomic changes. Among the detection and genotyping tools for *Paracoccidioides* spp. available since 2000 are PCR/DNA sequencing, conventional PCR, nested PCR, PCR-RFLP, qPCR, LAMP, and ISH/FISH [116–119]. Amplified fragment length polymorphism has recently emerged as a powerful molecular tool for typing *Paracoccidioides* species because of its ability to detect polymorphisms relevant to the epidemiology of PCM [32,120]. Duplex PCR and qPCR using single-tube multiplex probes are now available and offer high specificity for detecting and identifying members of the *P. brasiliensis* complex and *P. lutzii* in paraffin-embedded tissue samples and soil [31,119]. Combining these molecular tools allows the detection of *Paracoccidioides* species and species complexes, reducing the time of conventional diagnosis in this neglected disease [31]. Currently, next-generation sequencing, Nanopore sequencing, PacBio sequencing, and sequencing by expansion have limitations with respect to its application, including cost, accessibility, and challenges in data interpretation [121].

The application of immunoproteomics and bioinformatics in *Paracoccidioides* research has yielded encouraging results for identifying novel diagnostic candidates. Presently, substantial proteomic data are available; for example, comparative immunoproteomic studies using patient sera identified distinct sets of highly immunoreactive proteins during infection with *P. lutzii* (16 proteins, revealing isoforms of enolase as major antigens) and *P. brasiliensis sensu strictu* (25 proteins). These analyses revealed numerous previously undescribed antigens, including seven uncharacterized proteins specific to these species [122]. Complementary studies have further expanded the list of potential targets (e.g., 79 targets with general biological functions) and mapped the main antigenic B-cell epitopes for many of these proteins. This detailed epitope mapping is crucial for enabling the specific identification of different *Paracoccidioides* species or complexes [123,124].

A promising test integrates polymerase chain reaction (PCR) doubly labeled with biotin and digoxigenin with specific primers for the *GP43* and *GP70* genes, along with a paper-based lateral flow immunochromatographic (LFA) assay with carbon nanoparticles as a signal-generating system. The reaction can be performed in portable thermocyclers and visualized with LFAs, providing rapid results. This test detects amplicons of 0.21 or 0.10 ng, depending on whether the results are evaluated visually or with a smartphone. It is a rapid, cost-effective, and easy-to-use diagnostic method suitable for outpatient infection screening [125].

### Therapeutic and prophylactic strategies

Therapeutic and prophylactic strategies in PCM remain subjects of debate, encompassing the choice of antifungal compounds, management of sequelae, optimal treatment duration, strategies to improve patient compliance, routine monitoring of cell-mediated immunity, and the development of novel antifungal agents, vaccines, and immunotherapies. The Brazilian guidelines for PCM treatment recommend ITC, a first-line drug for patients with mild to moderate forms of PCM, and TMP-SMZ, also known as CMX, as a second-line treatment. AmB is recommended for severe cases [4]. However, the efficacy of ITC and TMP-SMZ has been demonstrated in the treatment of severe cases of acute/subacute and chronic forms [129], thus allowing AmB to be reserved for critical cases or those with intense organic involvement. Patient compliance is the greatest problem, since initial clinical improvement leads to treatment abandonment and an adherence of 45% [130]. A specific study revealed that the average duration of clinical cure was eight months, the average duration of serological cure was 17 months, and the average duration of treatment was 20 months. The time for clinical and serological cure was the same for the acute/subacute form, but was shorter for ITC than for TMP-SMZ in the chronic form [129].

***Treatment duration*.** PCM patients receive treatment for a long time, approximately two years, leading to low compliance, treatment discontinuation, and recovery. On the basis of experience in the treatment of cryptococcal meningitis in AIDS patients [131], the use of a single high-dose liposomal AmB and then daily ITC for a time to be determined and the association of antifungal compounds with recombinant cytokines could be evaluated.

***Cell-mediated immunity follow-up**.* Since a fraction of fungal cells may persist despite efficacious treatment, the restoration of cell-mediated immunity is essential to to restrain fungal survival in the quiescent stage state and prevent reactivation. Immunological monitoring has been carried out by observing decreasing antibody serum levels via immunodiffusion tests, which are based on correlations with the concentrations of IL-2 (increase), IFN-γ (increase), and IL-10 (decrease) in cell culture [132]. Consequently, direct assessment of cellular immunity is necessary to facilitate the medical decision to appropriately discontinue treatment. In this context, some immunological analysis approaches have been developed using pools of conserved peptides from the genus *Paracoccidioides* and whole blood to search for biomarkers [133].

***Drug repositioning and new antiparacoccidioidal compounds*.** Although resistance to antifungal agents currently used in PCM treatment has rarely been reported, several challenges remain, including poor drug penetration into the CNS, drug interactions—especially with treatments for comorbidities—and adverse effects. In this context, drug repositioning has emerged as a promising and cost-effective strategy for identifying new therapeutic alternatives, including agents with both antifungal properties and potential efficacy against pulmonary fibrosis [134,135]. In parallel, the identification of novel compounds that target fungal-specific pathways is also urgently needed. Rational drug design on the basis of molecular targets has led to the identification of ten potential candidates, with emphasis on thioredoxin reductase (TrxRr1), sterol 24-C-methyltransferase (Erg6), and alpha-1,2-mannosyltransferase (Kre2) [136]. Recent *in silico* screening identified small molecules with inhibitory activity against these targets, some of which have shown promising antifungal effects in preclinical studies. Among them, two 1,3,4-oxadiazole derivatives (LMM5 and LMM11) significantly reduce the fungal burden in the lungs of infected animals, with efficacy comparable to that of ITC [137]. Notably, LMM6, another 1,3,4-oxadiazole derivative with a chemical structure very similar to LMM11, exhibited superior results (patent BR132020004201E2). This molecule primarily induces necrosis and interferes with oxidative stress [104]. Together, these advances reinforce both rational design and drug repurposing as synergistic strategies to expand the antifungal arsenal against PCM.

***Antifungal and host-directed therapy*.** Another approach is to focus on the positive interaction between antifungal drugs and the immune system of the host via a combination of antifungal and host-directed therapies. For example, in experimental models, antifungal treatment with ITC not only directly reduces fungal burden but also modulates host immunity. ITC promotes the activation of polymorphonuclear neutrophils with enhanced production of reactive oxygen species, thereby strengthening innate antifungal defenses. Moreover, it decreases Th2 cytokines while favoring Th1/Th17 responses, ultimately skewing adaptive immunity toward a protective profile [138]. In addition, antifungals have positive effects on the immune system, leading to better outcomes in experimental PCM.

***Vaccines and immunotherapy*.** Vaccines, along with antifungal drugs and immunotherapies, represent promising clinical strategies for reducing the fungal burden in PCM. Importantly, these vaccines induce a protective antigen-specific immune response with a Th1/Th17 profile and stimulate the production of *Paracoccidioides*-specific IgG2a antibodies. Experimental studies in PCM have explored various vaccine platforms, including attenuated, inactivated fungal cells; recombinant/subunits; DNA-based vaccines; vaccines conjugated to nanoparticles; cellular therapies using primed dendritic cells; and immunobiological therapies, including monoclonal antibodies. Moreover, these vaccines have been evaluated in both prophylactic and therapeutic strategies. Among the various molecules tested over the past decades, peptide P10 has been extensively studied across multiple platforms and strategies, with highly promising results [139–141].

### Social aspects

PCM significantly impacts not only the physical health of affected individuals but also their social well-being. The prevalence of PCM primarily affects rural male workers in Central and South America, with a strong correlation with social determinants of health, such as poverty and limited access to healthcare services. Delayed diagnosis and treatment lead to severe disease outcomes, often resulting in irreversible sequelae such as pulmonary fibrosis, emphysema, vocal impairment, and disfiguring skin lesions [51,142]. These complications severely limit patients’ ability to return to work, leading to premature retirement, economic dependency, and social isolation. Moreover, the chronic nature of PCM and its debilitating effects contribute to social stigma and psychological distress, which often manifests as anxiety, depression, and reduced quality of life [143]. Despite the significant socioeconomic and mental health impacts, PCM remains an understudied issue regarding standardized quality-of-life and disability assessments [142,144]. Recent studies emphasize the urgent need to incorporate validated assessment tools, such as the WHO Quality of Life Brief Version (WHOQOL-BREF), the 36-item Short-Form Survey (SF-36), and condition-specific tools such as St. George’s Respiratory Questionnaire [143]. The implementation of such tools could greatly increase our understanding of the socioeconomic and mental health consequences of PCM, facilitating the development of more effective public health strategies, tailoring interventions, and improving the allocation of resources to address the health, rehabilitation, and social reintegration needs of affected populations.

## Conclusion

In the 21st century, substantial progress has been made in understanding and managing PCM. However, the disease remains neglected, with persistent gaps in public health recognition, notification, and clinical management. Epidemiological shifts driven by environmental changes, agricultural expansion, and socioeconomic factors have underscored the need to strengthen surveillance systems. Recent taxonomic revisions of *Paracoccidioides* spp. highlight the need for standardized diagnostic and reporting protocols. Advances in diagnostic technologies, improved understanding of fungal‒host interactions, and ongoing therapeutic developments have improved disease management. However, challenges related to therapy persist, including the limited availability of antifungal options and complex drug interactions. Addressing the significant social impacts of PCM, including stigma, economic hardship, and impaired quality of life, requires integrated approaches incorporating social rehabilitation, standardized disability assessments, and advocacy to incorporate PCM as a notifiable disease in public health policies. To address the aforementioned challenges and enhance outcomes for affected individuals, it is imperative that sustained international collaboration and multidisciplinary research efforts be undertaken. Furthermore, there is a need for solid and continuous public investment policies and training for health teams that can contribute to mitigating or removing this important disease from its neglected status.

## Supporting information

S1 File Consensus process for the classification of PCM endemicity in the Americas. (PDF)

S2 File Mycological examinations for the diagnosis of paracoccidioidomycosis (PCM). **(A)** Direct mycological examination of a cervical lymph node biopsy from a PCM patient showing round, birefringent, budding cells. **(B)** Direct mycological examination with lactophenol from a *Paracoccidioides* culture (37°C) showing round, birefringent, multibudding cells. **(C)** Cerebriform culture of *Paracoccidioides brasiliensis* on Sabouraud medium (37°C). Figures kindly provided by Gustavo Giusiano.
(PPTX)

S3 File Clinical manifestations of paracoccidioidomycosis (PCM). **(A)** Patient with the chronic form of PCM presenting a vegetative-verrucous lesion approximately 2.0 cm in diameter, located on the nasal dorsum. **(B)** Patient with the chronic form of PCM presenting an ulcerated lesion with a granular base and hemorrhagic dots, extending from the lip commissure to the buccal mucosa, characteristic of Aguiar-Pupo’s moriform stomatitis. **(C)** Patient with the acute form of PCM showing anterior and posterior cervical and submandibular lymphadenomegaly with inflammatory signs. Figures kindly provided by Ricardo de Souza Cavalcante.
(PPTX)

S1 Box Key learning points. (DOCX)

S2 Box Top five papers. (DOCX)
