# Supplementary material for: Paracoccidioidomycosis in the 21st century: Challenges and milestones
Source: PLoS Negl Trop Dis. 2026 Jan 6;20(1):e0013819. doi: 10.1371/journal.pntd.0013819 (PMC12774349; doi:10.1371/journal.pntd.0013819)
Supplement: S1 File — (PDF) [file pntd.0013819.s001.pdf]

Consensus process for the classification of PCM endemicity in the Americas

The endemicity map presented in Figure 1 was constructed using a modified Delphi consensus approach [1], adapted to the context of an international scientific meeting. The process was carried out during the International Conference on Paracoccidioidomycosis (PCM XXI) held in December 2024 (Campo Grande, Brazil), with the participation of clinical and epidemiological experts from several endemic countries.

The Delphi method is a structured and iterative consensus technique that relies on the judgment and expertise of specialists, being widely applied in epidemiological surveillance, particularly when formal notification systems or reliable incidence data are unavailable. This characteristic makes it especially suitable for PCM, a neglected disease that is not a notifiable condition in any Latin American country.

During dedicated sessions, experts reviewed available epidemiological data (published studies, hospital case series, and surveillance reports) and complemented them with unpublished local observations. Through structured discussions and interactive rounds of debate, consensus was reached on the classification of endemicity levels (high, moderate, low, sporadic) for different regions of the Americas.

This consensus-based approach ensured that the final map integrated both published evidence and the practical experience of experts directly involved in PCM management across endemic areas.

Reference

[1] Shang Z. Use of Delphi in health sciences research: A narrative review. Medicine (Baltimore). 2023;102:e32829. doi:10.1097/MD.0000000000032829.
