# Supplementary figures and images for: Paracoccidioidomycosis in the 21st century: Challenges and milestones
Source: PLoS Negl Trop Dis. 2026 Jan 6;20(1):e0013819. doi: 10.1371/journal.pntd.0013819 (PMC12774349; doi:10.1371/journal.pntd.0013819)

## Slide 1
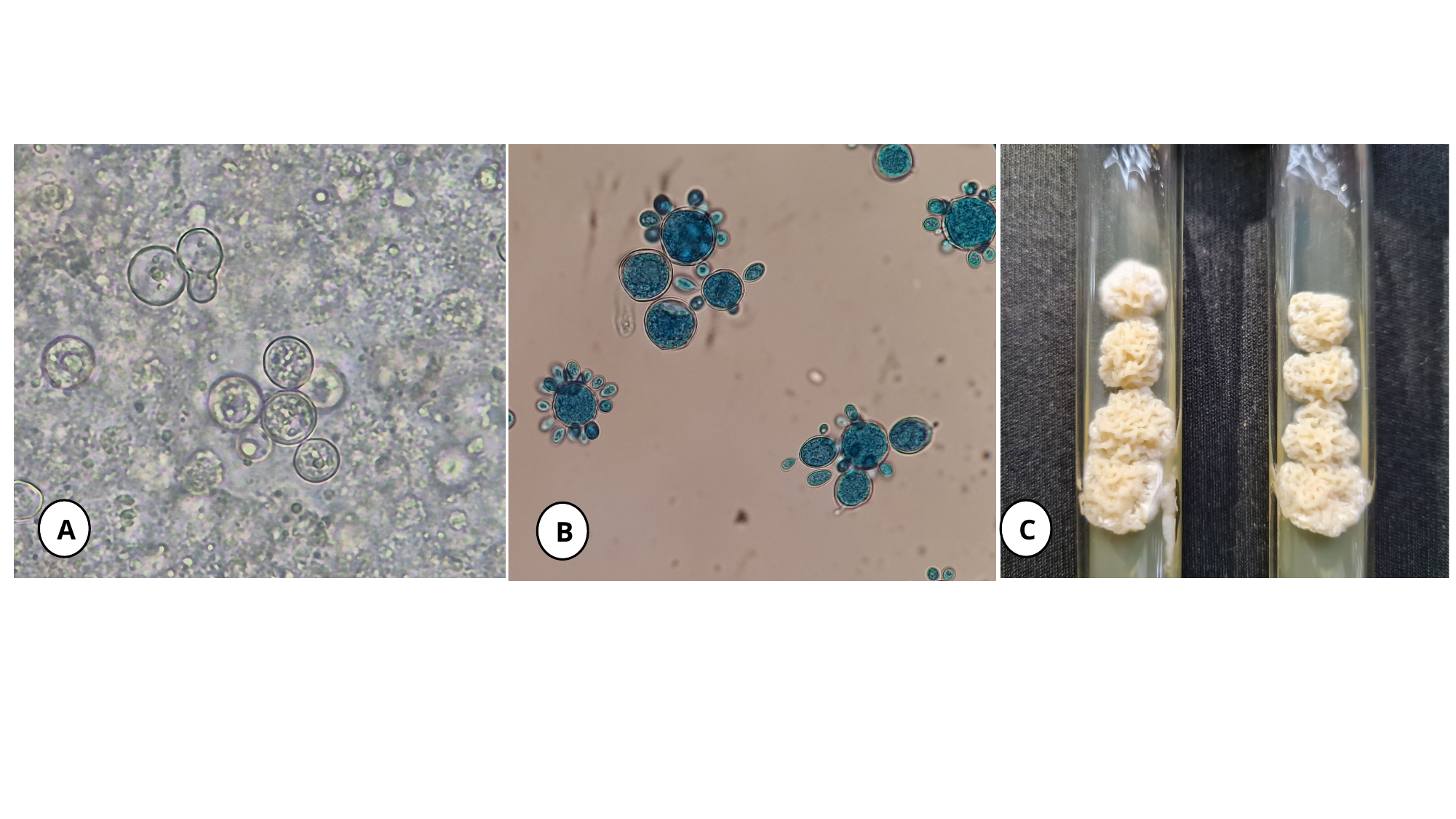

C
A
B

Supplement: S2 File — (A) Direct mycological examination of a cervical lymph node biopsy from a PCM patient showing round, birefringent, budding cells. (B) Direct mycological examination with lactophenol from a Paracoccidioides culture (37°C) showing round, birefringent, multibudding cells. (C) Cerebriform culture of Paracoccidioides brasiliensis on Sabouraud medium (37°C). Figures kindly provided by Gustavo Giusiano. (PPTX) [file pntd.0013819.s002.pptx]

## Slide 1
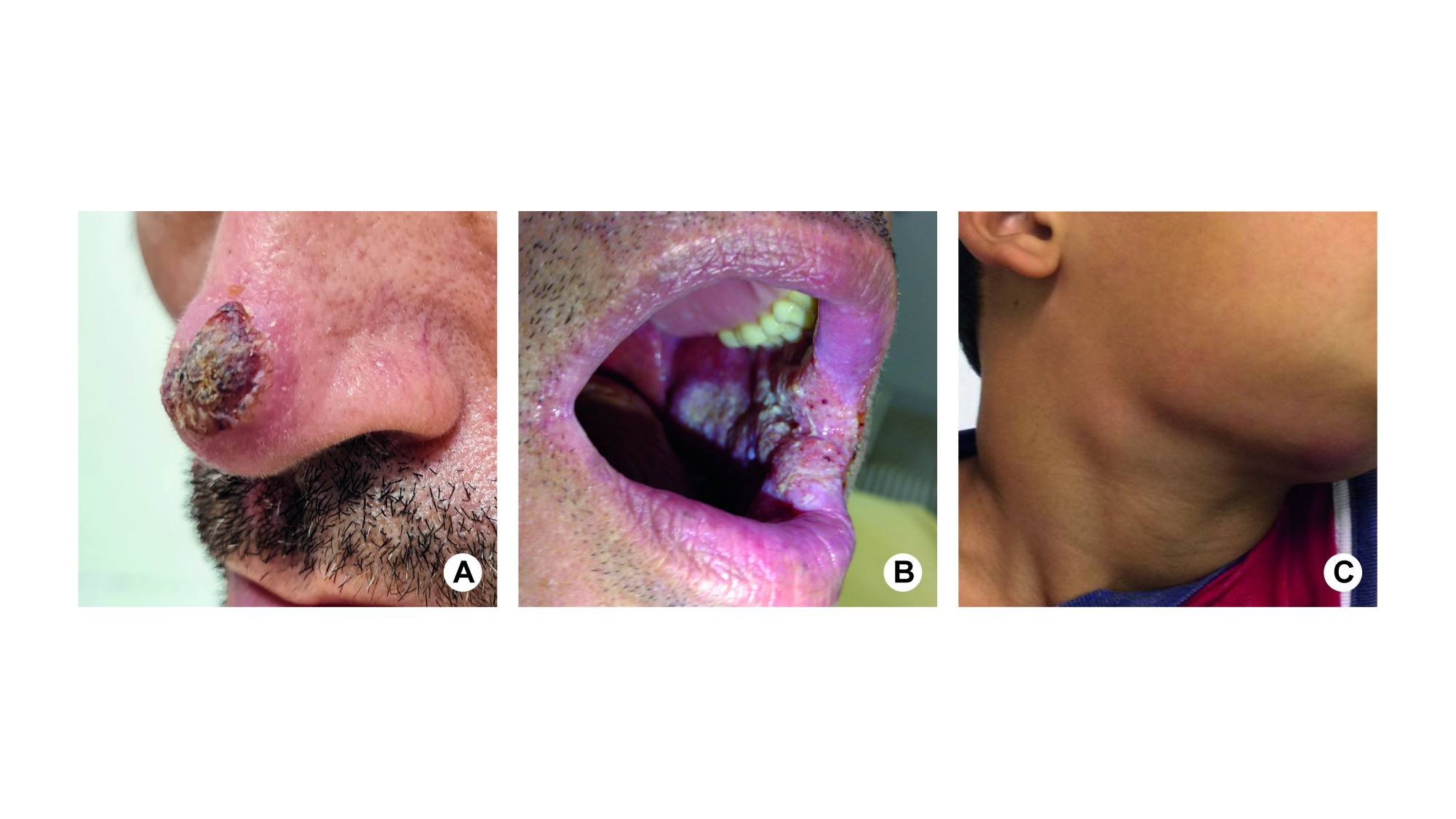

Supplement: S3 File — (A) Patient with the chronic form of PCM presenting a vegetative-verrucous lesion approximately 2.0 cm in diameter, located on the nasal dorsum. (B) Patient with the chronic form of PCM presenting an ulcerated lesion with a granular base and hemorrhagic dots, extending from the lip commissure to the buccal mucosa, characteristic of Aguiar-Pupo’s moriform stomatitis. (C) Patient with the acute form of PCM showing anterior and posterior cervical and submandibular lymphadenomegaly with inflammatory signs. Figures kindly provided by Ricardo de Souza Cavalcante. (PPTX) [file pntd.0013819.s003.pptx]
