# Supplementary material for: Paracoccidioidomycosis in the 21st century: Challenges and milestones
Source: PLoS Negl Trop Dis. 2026 Jan 6;20(1):e0013819. doi: 10.1371/journal.pntd.0013819 (PMC12774349; doi:10.1371/journal.pntd.0013819)
Supplement: S1 Box — (DOCX) [file pntd.0013819.s004.docx]

> Paracoccidioidomycosis (PCM) epidemiology is dynamic and strongly influenced by anthropogenic and environmental changes.

> Mandatory notification of PCM is essential across all endemic countries in Latin America to enhance surveillance, guide public health interventions, and reduce disease burden.

> The identification of cryptic species within the *Paracoccidioides* genus along with limitations of current diagnostic methodologies underscores the need for standardized, sensitive, and accessible diagnostic tools tailored to species diversity.

> Sequelae, particularly pulmonary fibrosis and functional disability, remain significant long-term challenges, emphasizing the necessity for clinical trials combining antifungal and antifibrotic agents.

> The social and psychological impacts of PCM have been overlooked, emphasizing the need for disability assessments and rehabilitation in care strategies.
