# Supplementary material for: Paracoccidioidomycosis in the 21st century: Challenges and milestones
Source: PLoS Negl Trop Dis. 2026 Jan 6;20(1):e0013819. doi: 10.1371/journal.pntd.0013819 (PMC12774349; doi:10.1371/journal.pntd.0013819)
Supplement: S2 Box — (DOCX) [file pntd.0013819.s005.docx]

1. Shikanai-Yasuda MA, Mendes RP, Colombo AL, Queiroz-Telles F de, Kono ASG, Paniago AM, et al. Brazilian guidelines for the clinical management of paracoccidioidomycosis. Rev Soc Bras Med Trop. 2017; 0. doi:10.1590/0037-8682-0230-2017

2. Martinez R. New trends in Paracoccidioidomycosis epidemiology. J Fungi (Basel). 2017;3: 1. doi:10.3390/jof3010001

3. Teixeira MM, Theodoro RC, de Carvalho MJA, Fernandes L, Paes HC, Hahn RC, et al. Phylogenetic analysis reveals a high level of speciation in the Paracoccidioides genus. Mol Phylogenet Evol. 2009;52: 273–283. doi:10.1016/j.ympev.2009.04.005

4. Giusiano G, Aguirre C, Vratnica C, Rojas F, Corallo T, Cattana ME, et al. Emergence of acute/subacute infant-juvenile paracoccidioidomycosis in Northeast Argentina: Effect of climatic and anthropogenic changes? Med Mycol. 2019;57: 30–37. doi:10.1093/mmy/myx153

5. Cavalcante R de S, Sylvestre TF, Levorato AD, de Carvalho LR, Mendes RP. Comparison between itraconazole and cotrimoxazole in the treatment of paracoccidiodomycosis. PLoS Negl Trop Dis. 2014;8: e2793. doi:10.1371/journal.pntd.0002793
